# Determination of Displacement Fields at the Sub-Nanometric Scale

**DOI:** 10.3390/ma12111804

**Published:** 2019-06-03

**Authors:** Cesar A. Sciammarella, Federico M. Sciammarella, Luciano Lamberti

**Affiliations:** 1 Department of Mechanical, Materials and Aerospace Engineering, Illinois Institute of Technology, Chicago, IL 60616, USA; sciammarella@iit.edu; 2 Department of Mechanical Engineering, Northern Illinois University, DeKalb, IL 60115, USA; sciammarella@niu.edu; 3 Dipartimento di Meccanica, Matematica e Management, Politecnico di Bari, Bari 70126, Italy

**Keywords:** sub-nanometric continuum kinematic fields, strain fields, dislocations, 4HSiC crystals

## Abstract

Macroscopic behavior of materials depends on interactions of atoms and molecules at nanometer/sub-nanometer scale. Experimental mechanics (EM) can be used for assessing relationships between the macro world and the atomic realm. Theoretical models developed at nanometric and sub-nanometric scales may be verified using EM techniques with the final goal of deriving comprehensive but manageable models. Recently, the authors have carried out studies on EM determination of displacements and their derivatives at the macro and microscopic scales. Here, these techniques were applied to the analysis of high-resolution transmission electron microscopy patterns of a crystalline array containing dislocations. Utilizing atomic positions as carriers of information and comparing undeformed and deformed configurations of observed area, displacements and their derivatives, as well as stresses, have been obtained in the Eulerian description of deformed crystal. Two approaches are introduced. The first establishes an analogy between the basic crystalline structure and a 120° strain gage rosette. The other relies on the fact that, if displacement information along three directions is available, it is possible to reconstruct the displacement field; all necessary equations are provided in the paper. Remarkably, the validity of the Cauchy-Born conjecture is proven to be correct within the range of observed deformations.

## 1. Introduction

Recently the authors [1,2,3,4] have carried out studies on the experimental mechanics determination of displacements and their derivatives at the macro and microscopic scales. Both fields were compared using the concept of representative volume element (RVE). In this paper, basic approaches to continuum mechanics are extended to the sub-nanometric level. Mechanics of materials covers multiple scale problems, depending on the type of the issues that are investigated. At each level exists an RVE that provides answers to posed questions. One very important relationship in the multiscale level analysis is the connection of the macro-analysis with the discontinuous nature of matter. Since the 19th century, efforts have been made to relate the macro-behavior of materials with the interactions between atoms and molecules. It was Augustin Louis Cauchy [5] the first author that introduced a mathematical model connecting the behavior of solids with their atomic structure. This initial analysis of the relationship between the continuum assumption and the discontinuous nature of solids is currently known as Cauchy-Born rule [6]. In the current form, the Cauchy-Born rule states that for small strains the position of atoms in a deformed crystalline medium can be predicted by the kinematics and dynamics of continuum mechanics in the range of small deformations and rotations. As it is going to be shown later on in this paper, the analysis of the relationship between atom positions and continuum displacement fields can be handled by extending Cauchy-Born rule from the small rotations and deformations to the range of deformations and rotations observed in crystalline metals. A very important role in this development is the concept of dislocation. Interestingly, this concept was introduced from a purely mathematical point of view, analyzing the unicity of the displacement function in the kinematics of the continuum. Volterra [7] in 1907 introduced the concept of dislocation, and later it was extended by Somigliana [8] and Love [9]. Volterra developed a geometrical type of dislocation that today is utilized in the mechanics of fracture, mode 1 utilized fracture mechanics. This abstract mathematical concept was adopted in the field of mechanics of materials to explain discrepancies between theoretical predictions and experimental measurements of the strength of crystals, by Taylor [10], Orowan [11], Polanyi [12] and Nabarro [13]. A new discipline was thus developed [14], the mathematical theory of dislocations that analyzes the interactions of the inner components of matter in the processes of deformation beyond the kinematics of small deformations and rotations of continuum mechanics. A considerable amount of literature has been developed to deal with the relationship between the discrete nature of matter and the continuum approach [15,16,17,18,19,20].

In order to validate the theoretical foundation of the concept of dislocations, it is necessary to have experimental verification of the existence of dislocations in crystalline arrays. This experimental evidence of dislocations in crystalline structures came in the 1960s thanks to the advancement in imaging technology. Images of edge dislocations in a crystalline structure were obtained through X-ray photogrammetry, using X-rays recordings and an optical reconstruction. In the 1970s, transmission electron microscopy provided a powerful tool to observe the structure and the deformations of crystalline matter. TEM techniques have been progressively improved over the years. Just to limit the survey to the last 15 years, References [21,22,23,24,25,26,27] report interesting examples of applications of TEM techniques to the detection of dislocations, assessment of strain and stress fields in crystals, nanowires and other nano-structures as well as attempts to improve the resolution of TEM patterns.

This paper utilizes the methodology described in References [1,2,3,4] applied to images generated by high-resolution transmission electron microscopy (HRTEM). It will be proven possible to extract information from HRTEM patterns using the same methodology developed for moiré and speckle patterns in the field of visible optics. The experimentally observed atom configurations in the HRTEM images are utilized to draw important conclusions on the kinematics of crystalline arrays. This is a critical work, which enables a deeper understanding of the behavior of materials and can lead in the future to the prediction of failure at much earlier stages in the life cycle of a material. This may result in preventative measures as well as provide new guidelines in the manufacturing of metals.

## 2. Analysis of HRTEM Images

Analyzing the atomic structure is possible because the HRTEM images and the images obtained by interference microscopes [28] are similar. An ideally plane wave electron beam interacts with the crystalline structure and produces an image at the image plane. The image of the microscope is a scalar field of gray levels resulting from the recording by the microscope sensor of the arriving electromagnetic magnetic wavefronts. The arriving signal is a phasor **E**(r)e^ϕ(r)^, where **E**(r) is the amplitude and ϕ(r) is the phase, and the sensor only records the amplitude. The image intensity distribution of gray levels is a function of the microscope contrast transfer function [29]. This function defines the spatial resolution of the microscope. The spatial resolution of the gray levels depends on the instrument properties and on the coherence of the illuminating electron beam. The levels of gray are recorded by a raster of sensors similar to the sensors utilized in electronic cameras. Hence, image resolution depends on sensor size similarly to the case of electronic cameras. The experimentally measured distribution of gray levels can be corrected via software by modifying the contrast transfer function. This is equivalent to correcting optical transfer function in visible optics. The gray level density distribution is a 2D function since it corresponds to images captured on a plane. Maximum observed gray level densities occur at regions where the maximum electronic density is located. In turn, maximum electronic densities take place at the atomic nuclei. As such, observed gray level distributions can be utilized as tools to define atomic positions in 2-D.

Another important factor of image formation in transmission microscopes is the thickness of the specimens. Absorption of electrons in the crystal limits thickness of observed crystal specimens [28]. Electrons absorption is a function of the applied voltages and results in allowable thicknesses on the order of nanometers. In the images analyzed in this paper, the thickness of the specimen was on the order of 10 nm and the number of atomic planes in the specimen was approximately 30. The depth of focus of the electron microscope was large enough that some 3D features at different depths may appear in the 2D image [28].

Another observation concerning the spatial resolution of the 2D image is that in place of utilizing the spatial resolution predicted by the optical analysis of the microscope, the spatial definition criteria proposed in Reference [28] was adopted. Accordingly, spatial resolution is defined by the measurable minimum distances in the HRTEM image plane. The concept of minimum distance requires some additional explanation. The electron microscope image that was analyzed in this paper [30] was obtained with an HRTEM JEOL 4000 EX-TEM (JEOL USA Inc., Peabody, MA, USA) operating at 400 KeV [31]. The electron wavelength at this voltage was 0.00193 nm, i.e., on the order of 0.002 nm. According to the criteria of HRTM [28], with this wavelength of 0.002 nm, one should be able to measure distances of the order of 0.001 nm. However, the HRTEM JEOL 4000EX-TEM used for recording the image has a maximum classical spatial resolution of 0.14 nm [31], two orders of magnitude larger than the theoretical value. This rating is a consequence of the aberrations of the microscope that lead to the above calibrated resolution of the microscope. This is the reason that the “minimum distance that can be measured” criterion was introduced in Reference [28] as a definition for spatial resolution. For the same reason mentioned in Reference [28], the same criterion was applied in this paper. The bicubic spline interpolation of the images was utilized to increase the spatial resolution of the images.

## 3. Methods for Measuring Deformations in Crystals

We will briefly give a description of the methodology utilized in this study to measure displacements in crystals caused by applied loads. More details will be given later, while at this point the main ideas only will be introduced. A detailed description of the applied methods implies a preceding description of the selected crystal specimen.

In experimental mechanics techniques that measure displacements and derivatives of displacements, two basic approaches are utilized. One approach is the direct measurements of distances changes through strain gages methods. The other approach is to apply tagged carriers, either deterministic or random carriers and through optical means create images of the carriers in a reference condition, and in a loaded condition. Then, the changes in the field are computed by the difference between reference configuration and loaded configuration. The scalar fields of gray levels are transformed in vectorial fields corresponding to deformations by either optical correlation generating fringes in the case of deterministic signals (moiré fringes) or by numerical correlation in the case of discrete image correlation (DIC).

In the analysis of a crystal, we have a deterministic signal, the crystal structure in the undeformed condition, and then the deformed structure caused by a loading process. The microscope produces an image of the atom positions in both configurations. It is possible to use also two approaches, measuring changes of distances in the crystalline configuration, utilizing basic parameters of the crystal atoms or a field approach applying fringe analysis techniques (for example, [1,2,3,4]). In the present study, the authors have selected a specimen to analyze a crystal that contained dislocations and experience deformations under the state of residual stresses produced by the presence of dislocations. The specimen was a thin crystal wafer of about 10 nm thickness under a state of plane-residual-stresses.

## 4. The 4HSiC Crystal

To understand the meaning of the analyzed images, it is necessary to deal with the crystallographic description of the observed crystal. In earlier publications, the authors [32,33,34] included some of the material reviewed in this section.

To obtain the images analyzed in this paper, two inches wafers were grown by vapor deposition oriented along the plane (0001) within ±0.5° of a SiC crystal. KOH etching at 500 °C or 510 °C was done on the Si (0001) faces of the wafers. Etch pits were observed by using a Nomarski interferometry while X-rays were used for observing the orientation of each pit array.

**Figure 1:**
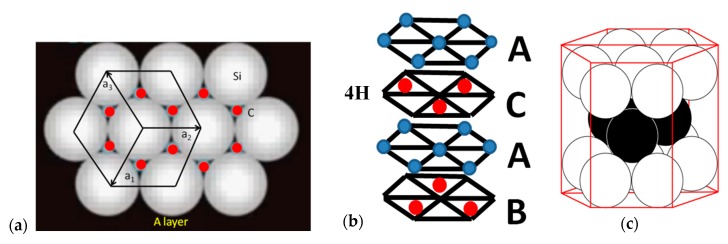
(**a**) Stacking sequence ACAB of 4HSiC crystal: red dots indicate the position of the following atomic layer shown in (**b**) at layers C and B; (**c**) 3D perspective of three initial layers.

The most common SiC polytypes have four and six SiC bi-layers to define unit cell repeating distance along c-axis [0001] directions. Figure 1 shows the ACAB sequence of 4HSiC crystal. The sequence is relative as radii of regions corresponding to each atom share the same magnitude. Hence, relative positions depend on possible geometrical arrangements of spheres. Figure 1a shows the arrangement denoted by the letter A, which assumes a sequence to be started by a Si atom. Stacking arrangement is sketched in Figure 1b while Figure 1c shows a perspective of the three initial layers.

**Figure 2:**
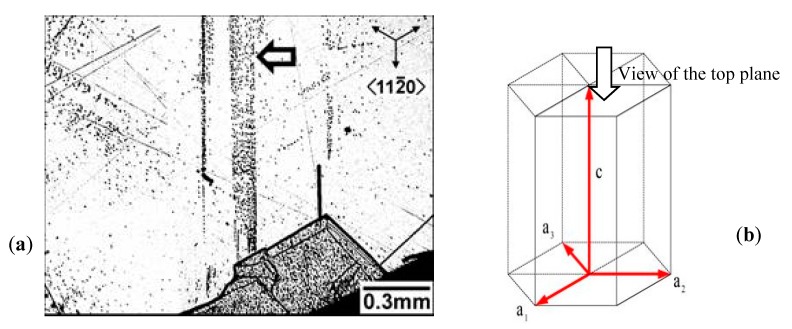
(**a**) Optical micrograph of etch pitch bands —(0001) face—of a 4HSiC wafer with bands along directions <11$\overset{-}{2}$0>; (**b**) theoretical structure of SiC hexagonal polytype with a_1_ = a_2_ = a_3_.

Figure 2a shows an optical micrograph of the etch pit bands —(0001) face— of a 4HSiC wafer. Bands run along directions <11$\overset{-}{2}$0> revealing the presence of threading edge dislocations. The theoretical structure of a SiC hexagonal polytype with a_1_ = a_2_ = a_3_ is shown in Figure 2b.

**Figure 3:**
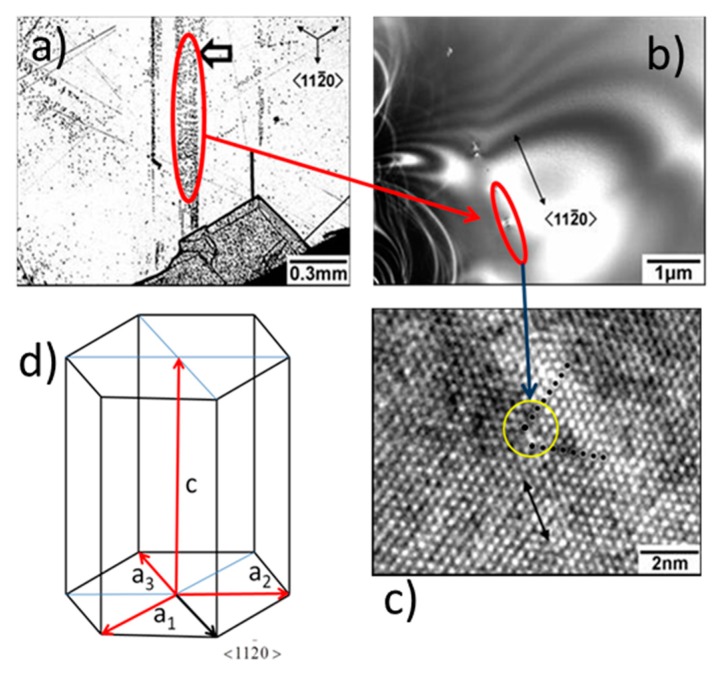
Dislocation gliding in the $<11\overset{-}{2}0>\{\overset{-}{1}100\}$ slip system during post-growth cooling: (**a**) Optical micrograph; (**b**) electron microscope image of red circled region; (**c**) HRTEM pattern of crystal (dark dots correspond to extra-atomic planes); (**d**) theoretical structure of the SiC crystal with a_1_ = a_2_ = a_3_ and dislocation’s Burgers vector oriented as a_3_.

Figure 2 shows the threading edge dislocation created by the cooling of SiC crystal. A misoriented crystal grew because of the presence of this dislocation (see Figure 2a). The high energy concentration in that region led to creating slip bands that caused the atomic plane to slide as shown in Figure 3a. The dislocation movement resulted in the generation of two extra half planes indicated by the black dots in Figure 3c to reduce the energy in the unit cell. This allowed crystal structure to be stabilized.

Figure 3c is the positive of a negative where the recorded levels of gray of maximum electron density are transformed back to recorded intensities. The dots have been added in the figure to indicate the presence of additional planes within the atomic arrangement. Figure 3 presents the optical micrograph of Figure 2a together with an electron microscope image of an etch pit (Figure 3b) and an HRTEM image around a threading dislocation in the array (see Figure 3c). Figure 3d shows that Burgers vectors of edge dislocations are of the type a/3 $<11\overset{-}{2}0>$; **a** is the 4HSiC lattice parameter vector.

### 4.1. Recovery of the Displacement Information from the TM Image

**Figure 4:**
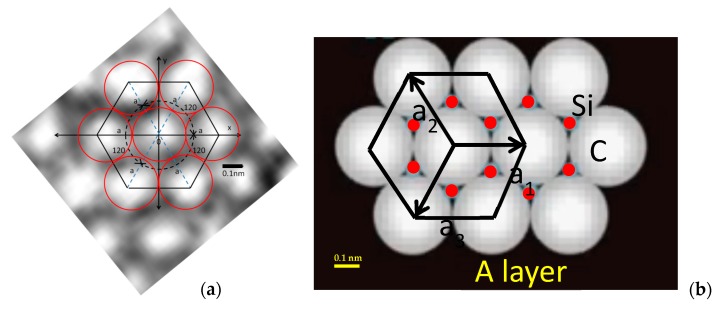
(**a**) Elementary cell of hexagonal packing identified in the HRTEM pattern resembling theoretical structure shown in (**b**).

In order to analyze the image content of the 2D rendering of the 3D stack of Figure 1, one can relate Figure 4, the intensity distribution of the 2D image, with the hexagonal elementary cell.

It can be concluded from visual inspection that the image of the atomic layer (Figure 4a), where the red circles are the atomic radii, is connected to the atomic arrangement of Figure 4b.

**Figure 5:**
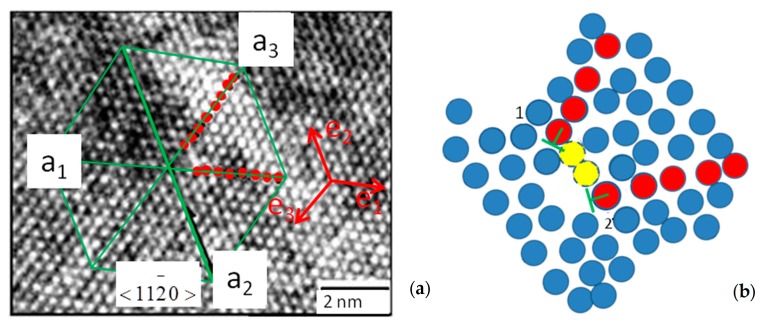
(**a**) Crystallographic directions $<11\overset{-}{2}0>$ ≡ **a**_2_; (**b**) Schematic representation of atomic positions in the neighborhood of edge dislocations (yellow dots are clarified later in the article).

A small squared region containing the elementary cell was cropped from the original image (Figure 3c), far from the dislocation region. Bicubic spline interpolation of pixels was performed to obtain the scale of Figure 5a. The red circles in the figure correspond to spheres of radius r = a/2 = 0.3073/2 = 0.1537 nm, one half of the SiC’s lattice parameter a = 0.3073 nm at 300 K. Using this parameter, the corresponding hexagon connecting centers of spheres can be sketched.

### 4.2. Study of the Changes in Elementary Cell Parameter in the Dislocation Region

Figure 5a illustrates the positions of crystallographic axes in the HRTEM pattern. Red dots correspond to the two extra atomic rows. Figure 5b schematically represents the dislocation region, including the position of the two edge dislocations generated by extra atomic planes. Deformation analysis of elementary cells relies on the idealization of a 3-D atomic array formed by superposing identical 2-D atomic arrays stacked in the vertical direction. Since this stack may change over depth because of the sequence shown in Figure 1 based in the covalent bonding of Si and C atoms, the present analysis is limited to the 2-D array.

The role of Fourier transform (FT) in recovering displacement information from gray levels recorded by a 2D sensor is discussed in detail in References [1,2,3,4]. The FT provides the components of projected displacement vectors corresponding to Cartesian coordinates x and y. This process requires the introduction of two orthogonal carriers in the case of Cartesian coordinates [1,2,3,4] that provide two projected displacements vectors that added give the final displacement vector. Due to the hexagonal structure of the crystal, the carriers are aligned in three different directions. It is necessary to point out that in 2D the tensors associated with the displacement field can be obtained if information on displacement in three different directions is provided.

**Figure 6:**
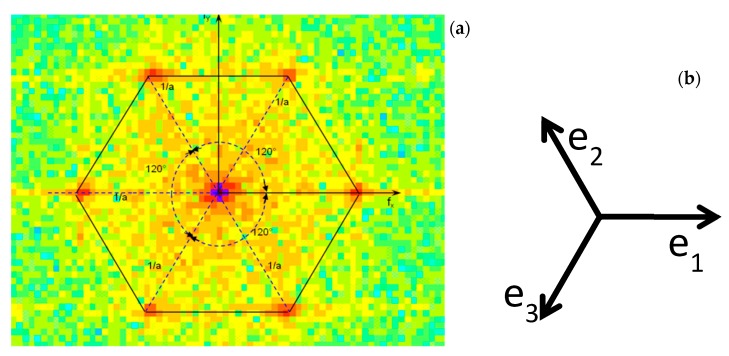
(**a**) FFT of the HRTEM pattern shown in Figure 6a; (**b**) reference system related to FFT.

Figure 6 shows the FFT of the crystal’s HRTEM pattern of Figure 3c. The direction f_x_ represents the family of crystallographic directions a1 = [1000] (see Figure 5a). The scale of Figure 6 is S_f_ = (0.0111) 1/nm per pixel. Figure 6 gives a statistical spatial average of frequency changes of elementary cells in the analyzed region. This means that Figure 6 is a graphical representation of the ensemble of the spatial frequencies of the RVE of the observed area. The hexagon shown in Figure 6 represents the average amplitude of **a** = 3.073 Å at 300 K. The changes in hexagon’s corners positions with respect to the average position indicated in Figure 6 reflect distortions of elementary cells caused by dislocations. Figure 4 represents the elementary undeformed cell for which the atomic vector **a** is constant. The **a** vector reflects the spatial configuration of atomic bonds binding together atoms. Since atoms continuously oscillate under thermal excitation at the T temperature, **a** is a statistical average over time. Figure 6 shows that the **a** vector is modified by the deformations caused by the dislocations. In the 2-D analysis, changes experienced by the elementary cell can be analyzed by superposing different changes in dimension and orientation of **a**.

In Figure 6b, there are indicated the versors corresponding to the geometrical orientation of the crystalline rows that correspond to the spatial changes introduced in the image of Figure 3c by rotating the image in such a way that corresponds to the orientation shown in Figure 4.

**Figure 7:**
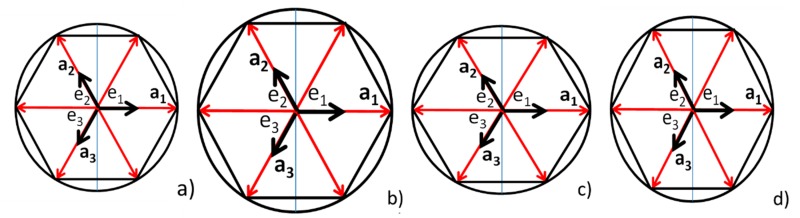
(**a**) Fundamental 4HSiC cell; (**b**) original cell; (**c**) elongated cell along the vertical direction (blue line); (**d**) shortened cell along the vertical direction.

The hexagon corresponding to the undeformed crystal is equilateral, meaning that all the sides have the same length, and it is also equiangular, meaning that the vertex angles are equal. In the display of the FFT in Figure 6a, the increase in the levels of red of the pixels indicate the predominant frequencies in the analyzed image. Figure 6 represents the ensemble of all the hexagons present in the analyzed image. The levels of intensity of the pixels represent the predominant shapes. It is possible to see that the main deviations from the original shape are of the order of a small number of pixels of lesser intensity (fewer in number). Therefore, applying the concept of ergodicity, we can represent the ensemble averages by the configurations shown in Figure 7.

A graphical interpretation of this observation is given in Figure 7. Figure 7a represents the basic unit cell. Figure 7b shows an expansion of the basic unit cell; this means that the parameter **a** of the undeformed unit cell has been expanded to become a unit cell representing an isotropic point of the strain and stress field. The opposite of these conditions is the contraction of the parameter **a**. Figure 7c shows another possible change of the unit cell, the parameter **a**_1d_ > **a**_1_, where the subscript “d” has been added to indicate the deformed condition and **a**_1d_ > **a**_2d_ = **a**_3d_ ≥ **a**_2_ = **a**_3_. Finally, Figure 7d shows the other alternative, **a**_1d_ < **a**_2d_ = **a**_3d_ ≥ **a**_2_ = **a**_3_. These different alternatives cover all possible states of the 2D plane strain conditions.

The experimental observations presented above confirm the validity of the Cauchy-Born rule expressed as ensemble configuration in the sense of representative volume rule applied to continuum kinematics.

## 5. Eulerian Coordinates Expression of Cauchy-Born Rule

As mentioned before, the elementary cell provides a system of reference along three directions that can be utilized as a strain gage rosette [35]. We will develop the necessary expressions for analyzing the deformation of elementary cells by equating them to three arms rosettes.

**Figure 8:**
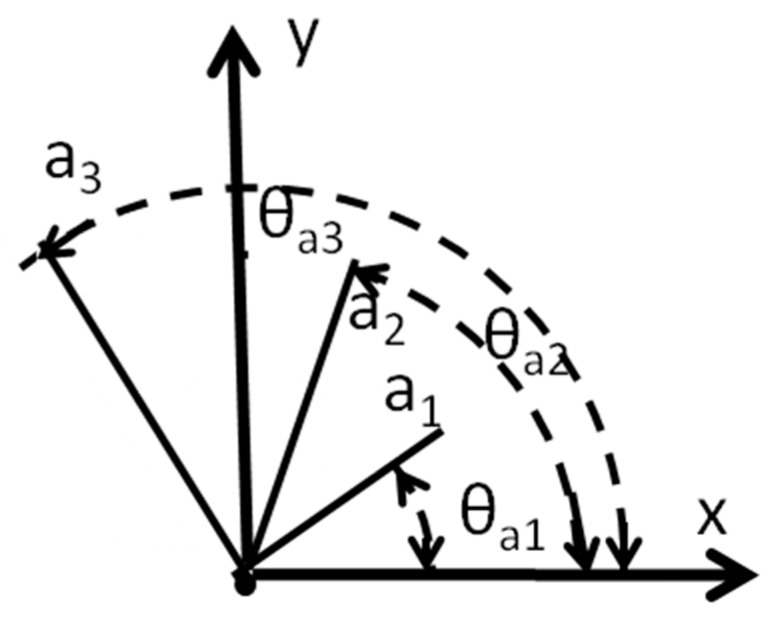
Relationship between displacement derivatives measured along three different versors.

Figure 8 shows the basic reference system including versors **a_i_**(I = 1,2,3). Positions of atoms in the deformed configuration in the hexagonal configurations can be described by a vectorial equation:

(1) $$u=u_{a1}a_{1}+u_{a2}a_{2}+u_{a3}a_{3}.$$

Displacement components along versors **a_1_**, **a_2_** and **a_3_** define actual sizes and orientations in Cartesian coordinates of parameters of the axes of a general local hexagon. Equation (1) corresponds to the main directions shown in Figure 6b. This figure provides the ensemble averages of the changes of frequencies of the main axis of the hexagon of the basic cell. The highest intensities in the corners of the hexagon of Figure 6a indicate the most predominant configurations that are around the theoretical parameter a = 3.073 Å of the SiC at 300 K. The length and angles of the vectors **a_i_** change, but these relative of changes of the parameters **a** are small quantities and the ensemble averages can be represented by the shapes indicated in Figure 7.

We can consider the 2D tensor of the derivatives in Cartesian coordinates [2],

(2) $$[J]=\left[\begin{array}{cc} \frac{\partial u}{\partial x} & \frac{\partial u}{\partial y}\\ \frac{\partial v}{\partial x} & \frac{\partial v}{\partial y} \end{array}\right].$$

Then taking the symmetric component of this tensor [4],

(3) $$[J_{s}]=\left[\begin{array}{cc} \frac{\partial u}{\partial x} & \frac{1}{2}\left(\frac{\partial u}{\partial y}+\frac{\partial v}{\partial x}\right)\\ \frac{1}{2}\left(\frac{\partial u}{\partial y}+\frac{\partial v}{\partial x}\right) & \frac{\partial v}{\partial y} \end{array}\right].$$

We can rotate the above tensor along the directions of the three versors **a_i_**,

(4) $$\left\{ \begin{array}{l} \frac{\partial u_{a1}}{\partial a_{1}}=\frac{\partial u}{\partial x}\cos^{2}\theta_{a1}+\frac{\partial v}{\partial y}\sin^{2}\theta_{a1}+\left[\frac{\partial u}{\partial y}+\frac{\partial v}{\partial x}\right]\cos\theta_{a1}\sin\theta_{a1}\\ \frac{\partial u_{a2}}{\partial a_{2}}=\frac{\partial u}{\partial x}\cos^{2}\theta_{a2}+\frac{\partial v}{\partial y}\sin^{2}\theta_{a2}+\left[\frac{\partial u}{\partial y}+\frac{\partial v}{\partial x}\right]\cos\theta_{a2}\sin\theta_{a2}\\ \frac{\partial u_{a3}}{\partial a_{3}}=\frac{\partial u}{\partial x}\cos^{2}\theta_{a3}+\frac{\partial v}{\partial y}\sin^{2}\theta_{a3}+\left[\frac{\partial u}{\partial y}+\frac{\partial v}{\partial x}\right]\cos\theta_{a3}\sin\theta_{a3} \end{array}\right..$$

From Equation (4), we obtain principal derivatives with respect to coordinate system x-y as well as the corresponding orientation of principal directions:

(5) $$\left\{ \begin{array}{l} \frac{\partial u_{1}}{\partial x}=\frac{\frac{\partial u}{\partial x}+\frac{\partial v}{\partial y}}{2}+\sqrt{{\left[\frac{\frac{\partial u}{\partial x}-\frac{\partial v}{\partial y}}{2}\right]}^{2}+{\left[\frac{1}{2}\left(\frac{\partial u}{\partial y}+\frac{\partial v}{\partial x}\right)\right]}^{2}}\\ \frac{\partial u_{2}}{\partial y}=\frac{\frac{\partial u}{\partial x}+\frac{\partial v}{\partial y}}{2}-\sqrt{{\left[\frac{\frac{\partial u}{\partial x}-\frac{\partial v}{\partial y}}{2}\right]}^{2}+{\left[\frac{1}{2}\left(\frac{\partial u}{\partial y}+\frac{\partial v}{\partial x}\right)\right]}^{2}} \end{array}\right.$$

(6) $$\tan2\theta =-\frac{\frac{\partial u}{\partial y}+\frac{\partial v}{\partial x}}{\frac{\partial u}{\partial x}-\frac{\partial v}{\partial y}}.$$

It should be noted that the above relationships define the Eulerian description of deformed crystal. The adopted reference system is attached to the crystal structure.

We have the equivalent of a three-elements rosette with arms at 0°, 120° and 240°. Calling **e_1_**, **e_2_** and **e_3_** the axes of the rosette, principal derivatives of displacement fields are determined as a function of measured derivatives along rosette axes, Equation (7):

(7) $$\frac{\partial u_{1}}{\partial x_{1}},\frac{\partial u_{2}}{\partial x_{2}}=\frac{1}{3}\;\left(\frac{\partial u_{e1}}{\partial e_{1}}+\frac{\partial u_{e2}}{\partial e_{2}}+\frac{\partial u_{e3}}{\partial e_{3}}\right)\;\pm \frac{\sqrt{2}}{3}\;\left[\sqrt{{\left(\frac{\partial u_{e1}}{\partial e_{1}}-\frac{\partial u_{e2}}{\partial e_{2}}\right)}^{2}+{\left(\frac{\partial u_{e2}}{\partial e_{2}}-\frac{\partial u_{e3}}{\partial e_{3}}\right)}^{2}+{\left(\frac{\partial u_{e3}}{\partial e_{3}}-\frac{\partial u_{e1}}{\partial e_{1}}\right)}^{2}}\right]$$

where x_1_ and x_2_ are the coordinate axes, e_1_≡x_1_ and the x_2_-axis is orthogonal to x_1_.

Principal directions of derivatives are determined as:

(8) $$\theta =\frac{1}{2}\mathrm{arctg}\left\{\frac{\sqrt{3}\left(\frac{\partial u_{e3}}{\partial e_{3}}-\frac{\partial u_{e2}}{\partial e_{2}}\right)}{2\frac{\partial u_{e1}}{\partial e_{1}}-\left(\frac{\partial u_{e2}}{\partial e_{2}}+\frac{\partial u_{e3}}{\partial e_{3}}\right)}\right\}.$$

## 6. Elementary Cell Analysis

**Figure 9:**
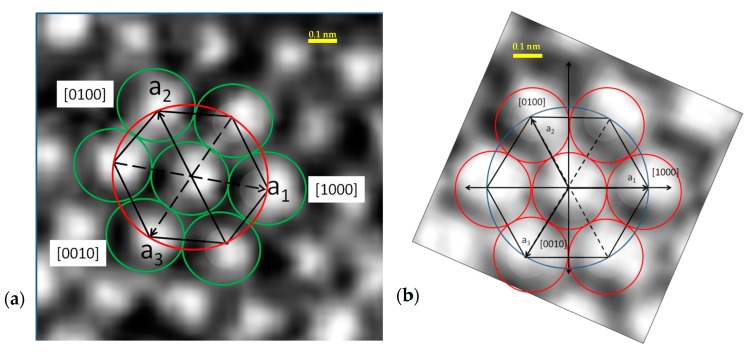
Elementary cells in the tension region (**a**) and compression region (**b**) of Figure 5c and Figure 6a.

After the development of the equations corresponding to the rosette analysis, it is interesting to analyze the obtained results shown in Figure 9a,b. The cell shown in Figure 9a is within the tension region of stress distribution generated by the dislocation. The axis **a_1_** is oriented in the crystallographic direction [1000]. Green lines limit the influence region of each atom and are no longer circles but ellipses. These lines fit the configuration of the elementary cell, which is contained in an ellipse very closely similar to the shapes of the green ellipses defining equilibrium positions of atoms. These ellipses are similar to those that in classical continuum kinematics define principal strains. The computed principal strains are: $\varepsilon_{1}^{E}$ = 0.0501, $\varepsilon_{2}^{E}$ = 0.0487, where the upper script “E” indicates the Eulerian strain. The principal direction $\varepsilon_{1}^{E}$ lays along crystallographic direction [1000] while the other principal direction $\varepsilon_{2}^{E}$ is perpendicular to it. Similar results were obtained using numerical methods based on molecular dynamics for the analysis of homogeneous states of deformation [17]. In 3-D, it can be assumed that the original spheres of Figure 4 become ellipsoids. Since initial isotropy of the field of one atom is broken, each elementary cell behaves as an anisotropic crystal (similar to photoelasticity).

Figure 9b shows an elementary cell of the compression region. Principal strains are as follows: $\varepsilon_{1}^{E}$ = −0.02550, coinciding with the direction [1000], and $\varepsilon_{2}^{E}$ = −0.0260, perpendicular to this direction. Interestingly, in the tension region, inter-atomic distances (black area) are more evident than for compression region. This reflects the asymmetry of the inter-atomic potential.

## 7. Elementary Cell Stresses

Applying the 120° rosette equations, principal strains at the level of elementary cell are determined. From measured principal values and utilizing mechanical properties of SiC, it is possible to compute principal stresses. The following values have been adopted, modulus of elasticity E = 402 GPa and Poisson’s ratio ν= 0.18. Applying the plane stress equations,

(9) $$\sigma_{p1}=\frac{E}{1-\nu^{2}}\left[\varepsilon_{p1}^{E}+\nu \varepsilon_{p2}^{E}\right]$$

(10) $$\sigma_{p2}=\frac{E}{1-\nu^{2}}\left[\varepsilon_{p2}^{E}+\nu \varepsilon_{p2}^{E}\right]$$

we obtained the results given in Table 1.

It is possible to see that the two fields, away from the dislocation, have states of almost constant isotropic stresses. The principal stress σ_p1_ is directed as [1000] while σ_p2_ is perpendicular to it.

## 8. Full Field Extension of Continuum Kinematics

**Figure 10:**
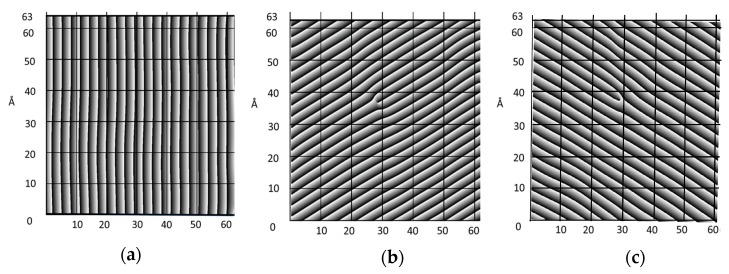
Wrapped phases of fringes corresponding to crystal structure: (**a**) **e**_1_; (**b**) **e**_2_; (**c**) **e**_3_.

In Section 4, it has been mentioned that the two approaches adopted in this paper to extract displacement information were the analysis of the elementary crystalline cell and a field approach. This section is devoted to the development of the second approach. Figure 10 is obtained from Figure 6a, filtering selected orders at the corner of the hexagon and computing the inverse FFT. A system of three fringe patterns at 120° of each other is obtained and in Figure 10 the wrapped phase patterns are plotted.

The orientation of the fringes in the space has been changed because the direction **e**_1_ has been made horizontal. As mentioned before, three systems of fringes at orientation 120° to each other can be extracted from the FFT of Figure 6a: this configuration is the equivalent of the three-arm rosette in the elementary cell analysis extended to the full field. The interpretation of the procedure to extract information from these patterns changes depends on adopted paths. One can follow the traditional interpretation based on the Cartesian coordinate system of reference. From the patterns of Figure 10, it is possible to obtain the necessary derivatives for getting components of the **J** tensor, Equation (2). Then, by utilizing Equations (2) through (8), one can compute the Euler-Almansi strain tensor components in the Eulerian description of the deformed continuum using in 2D the coordinate system x-y-z,

(11) $$\varepsilon_{x}^{E}=1-\sqrt{1-2\frac{\partial u}{\partial x}+{\left(\frac{\partial u}{\partial x}\right)}^{2}+{\left(\frac{\partial v}{\partial x}\right)}^{2}}$$

(12) $$\varepsilon_{y}^{E}=1-\sqrt{1-2\frac{\partial v}{\partial y}+{\left(\frac{\partial v}{\partial y}\right)}^{2}+{\left(\frac{\partial u}{\partial y}\right)}^{2}}$$

(13) $$\left(\varepsilon_{\mathrm{xy}}^{E}\right)=\arcsin\frac{\frac{\partial u}{\partial y}+\frac{\partial v}{\partial x}-\frac{\partial u}{\partial x}\frac{\partial u}{\partial y}-\frac{\partial v}{\partial x}\frac{\partial v}{\partial y}}{\left(1-\varepsilon_{x}^{E}\right)\left(\;1-\varepsilon_{y}^{E}\right)}.$$

**Figure 11:**
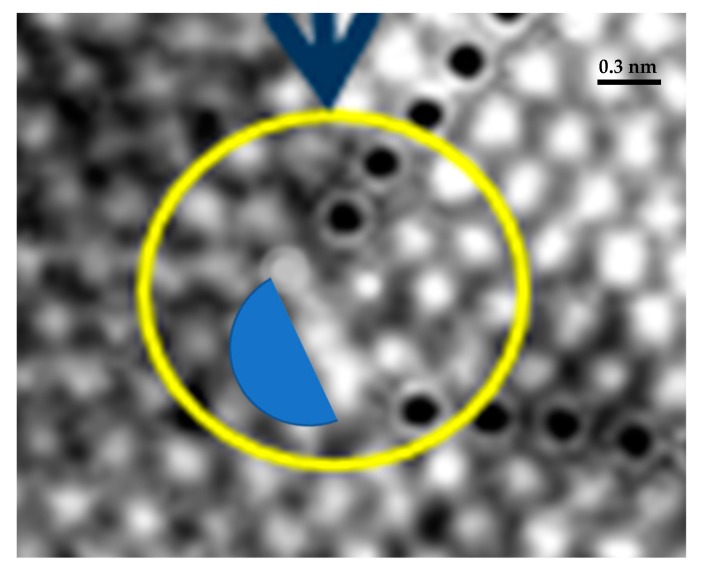
Enlargement of Figure 3c showing the dislocation region.

There is another alternative procedure based on the analysis of the hexagonal structure of the crystal, analyzing how this structure changes at different locations based on the changes of the parameters, a_i_, with I = 1, 2, 3. This procedure utilizes the continuum approach but follows the changes of the hexagonal structure in the field. To describe this alternative analysis, we will start with the image of the dislocation shown in Figure 3c and further illustrated in Figure 5a,b, see Figure 11.

**Figure 12:**
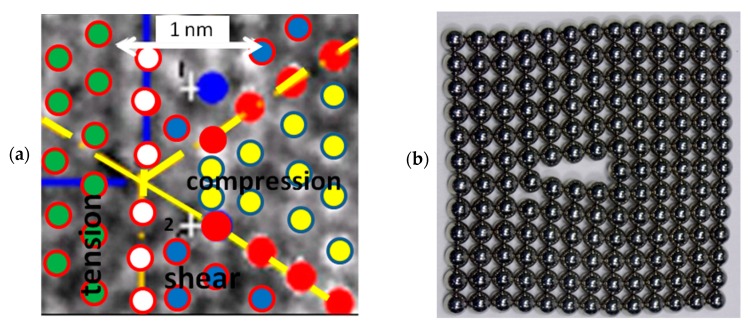
(**a**) Locations of atoms in the crystal region with the dislocation; (**b**) simulation of the presence of a dislocation using magnetic spheres [36].

In Figure 11, the black dots represent the two extra rows of atoms. The next row of atoms that follows the end of the dislocation rows shows a bright area where the position of the atoms is not well defined. The blue area that follows this row outlines a region where the light intensities indicate a cavity, area where there is not a discernible atomic structure. Figure 12a summarizes the preceding analysis; the yellow dots correspond to atoms that are under compression, the parameters **a**_i_ are reduced. The region including green circles is under tension as shown in Figure 9a: parameters **a**_i_ are increased. The region indicated as shear region corresponds to cases shown in Figure 7c,d.

The moiré fringe pattern phases of Figure 10 provide important information concerning the atomic arrangement. The kinematics of the continuum that is implicit in the isothetic lines (moiré fringes) can be utilized to determine inter-atomic spaces by means of digital moiré.

The concept of directional derivatives is used in this approach to the interpretation of patterns. The patterns of Figure 10a through Figure 10c are obtained from the FFT shown in Figure 6a. Figure 10a corresponds to the direction **e**_1_, Figure 10b corresponds to the direction **e**_2_ and Figure 10c to the direction **e**_3_. The fringes of the direction **e**_1_ do not contain a singularity, it is necessary to remember that the dislocations in fringe patterns do not necessarily overlap with a physical dislocation. The directions **e**_2_ and **e**_3_ show fringe dislocations that overlap with the physical dislocation. The pattern of Figure 10b displays the dislocation in coincidence with the direction of the physical dislocation. The fringes display displacements that are orthogonal to their directions and the directional derivatives display the rate of change of the displacements providing information on the deformation taking place in the corresponding directions. We are dealing with carrier fringes that correspond to the atomic arrangement, in this case, the pitch in the outlined directions is the distance **a** = 3.073 Å. To obtain the phases corresponding to the displacements from the phases displayed in Figure 10, it is necessary to subtract the phase of the undeformed carrier. Finally, through the differentiation of the patterns, one obtains the derivatives in the corresponding directions. Since we are dealing with a Eulerian representation and the local rotations are negligible, the directional derivatives provide the Eulerian strains,

(14) $$\varepsilon_{1}^{E}=\frac{\partial u_{e1}}{\partial e_{1}}$$

(15) $$\varepsilon_{2}^{E}=\frac{\partial u_{e2}}{\partial e_{2}}$$

(16) $$\varepsilon_{3}^{E}=\frac{\partial u_{e3}}{\partial e_{3}}.$$

**Figure 13:**
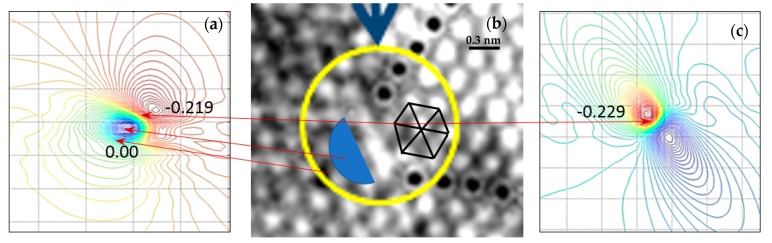
(**a**) Level lines of derivative ∂u_e2_/∂e_2_; (**b**) dislocation area; (**c**) level lines of derivative ∂u_e3_/∂e_3_. The spatial scale is Å.

These derivatives provide the changes in **a**_1_ and **a**_2_ and **a**_3_. Figure 13a shows the level lines of the derivative ∂u_e2_/∂e_2_. Figure 13b shows the image of the dislocation area. Figure 13c gives the level lines of the derivative ∂u_e3_/∂e_3_.

The level lines of derivative ∂u_e1_/∂e_1_ are almost constant in the field of view. Looking at Figure 13 and making a correlation between the image and the derivative plots, it is possible to visualize the connection between the rosette approach that was analyzed before and the fringe pattern approach. From the hexagon outlined in Figure 13 and from the directional derivatives, we obtain ∂u_e2_/∂e_2_ = 0.219 and ∂u_e3_/∂e_3_ = −0.229.

**Figure 14:**
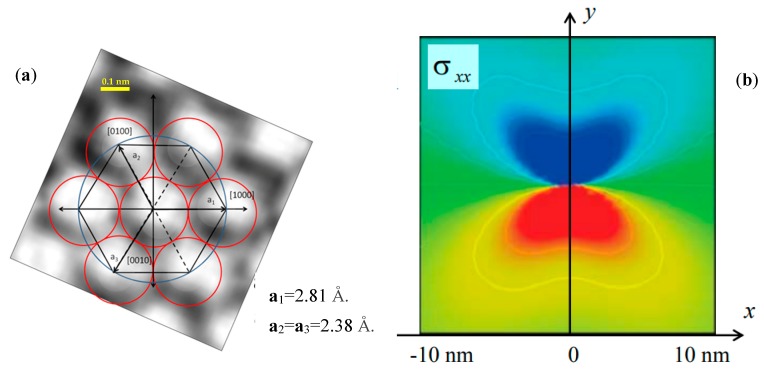
(**a**) Representative hexagon at the dislocation edge; (**b**) typical stress distribution σ_xx_ for a Volterra’s type dislocation [37]. The peak tensile stress (red) is 5 GPa; the peak compressive stress (blue) is −5 GPa.

Figure 14a shows the hexagon corresponding to the area of the dislocation edge showing a biaxial state of compression. The corresponding principal stresses can be computed as σ_p1_ = −108 MPa and σ_p2_ = −111.4 MPa.

Figure 14b shows the stress field of the normal stresses σ_xx,_ corresponding to aluminum, the material of the example for a Volterra’s type of dislocation [37]. It is interesting to observe that the distribution of stress levels for the Volterra’s type of dislocation is similar to the strain levels of the directional derivatives shown in Figure 13. One has in the neighborhood of the dislocation a half-plane that is compressed and the other half plane that is in tension. One solution corresponds to the Eulerian description and the deformations cannot be considered belonging to the region of small rotations and deformations. The other solution corresponds to the Lagrangian representation for infinitesimal deformation. Equilibrium conditions and geometry define displacement fields. Interestingly, application of Cauchy-Born rule as a homogenization technique gives a displacement field whose derivatives indicate the presence of a dislocation, thus merging with the theory of elasticity modeling of an edge dislocation.

Figure 12 and Figure 13 contain another interesting piece of information on atomic arrangements. Figure 12b shows the packing of rigid magnetic spheres [36]. However, the model of attracting rigid spheres used in crystallography literature to illustrate the concept of dislocation is misleading. In fact, this visualization does not describe the actual field of atomic arrangement for an edge dislocation. Atoms evidently cannot be modeled as rigid spheres: a cavity in the dislocation region of rigid spheres is about 6.5r_o_ large, where r_0_ represents the atomic radius, in this case, r_o_ = **a**/2 = 1.537 Å. In the actual dislocation, the depth is roughly about 4.736 Å, this is equivalent to 3.08r_o_, or about one half of the predicted value by the model of the rigid spheres. Indeed, the region near dislocations is not a cavity in the strict sense: in fact, the image reveals the presence of some electron density in the cavity. Cavities generated by the presence of crystalline structural defects have an interesting property that makes their presence experimentally detectable. Due to the low electronic density, if positrons are sent to this cavity, an electron and a positron form a pair. Since this pair is not stable, it decays into two γ-rays. Using a γ-rays detector, it is possible to count the number of events, which can be used for measuring the number of cavities present in a specimen [38].

## 9. Discussion and Conclusions

This paper is concerned with the utilization of state-of-the-art experimental mechanics methods to extend the concept of RVE volume of continuum mechanics at the atomic configuration level. Changes in the atomic configuration take place when the atomic equilibrium state is altered. These changes manifest themselves in the moiré patterns that can be obtained utilizing the material atoms as carriers of information. The work presented in this paper started with the purpose of getting experimental evidence supporting the Cauchy-Born conjecture on the relationship between changes in atomic configuration and continuum mechanics solutions as homogenizing functions of these changes in RVE volume of the crystalline material. This problem has two aspects, the kinematics of the continuum, a description of the motion of a medium, which has been analyzed in this paper. The other facet of the problem is the dynamics of the motion, not addressed in this paper. The presence of dislocations in a crystalline medium is the experimental approach used to get connections between Continuum Mechanics phenomenological description of the deformation of solids and the basic configuration changes occurring at the level of crystalline organization. Two approaches are utilized, the first is to take advantage of the hexagonal configuration of the basic unit cell of the material, SiC. An analogy between the basic unitary crystalline and a 120° rosette is established. This analogy is based on the fact that Figure 6a indicates that the majority of the elements present in the region of interest are distributed around the ideal hexagon. Basic changes in the configuration are monitored and they are observed in the compression area and in the tension areas of the specimen.

The second approach is based on the moiré patterns obtained by filtering Figure 6. A dual path in the analysis of the fringes has been introduced. Directional derivatives provide information concerning the elementary cell configuration. This approach yielded the configuration of an elementary cell adjacent to dislocations, Figure 13. The other approach is to take advantage of the fact that if one has displacement information along three directions one can reconstruct the displacement field; all the necessary equations are provided in the paper.

The validity of the Cauchy-Born conjecture has been proven to be correct. How far in the deformation range this assertion is correct? The digital moiré applied to the dislocation area shows that, in the sense of RVE, continuum mechanics variables can be utilized to predict actual dislocation kinematic variables. In conclusion, continuum mechanics is a powerful tool that can predict in the statistical sense, as a homogenization tool, the behavior of a metal crystalline structure under general deformation conditions.

## Figures and Tables

**Table 1:** Principal stresses computed for the deformed crystal.

	Tension (MPa)	Compression (MPa)
σ_p1_	23.00	−12.52
σ_p2_	22.87	−12.69
